# CHASE (Children’s Housing Assessment for a Safe Environment): a protocol for the inspection and modification of injury risks in children’s homes

**DOI:** 10.1186/s40621-023-00460-7

**Published:** 2023-10-11

**Authors:** Elise Omaki, Brendan Brown, Isabel Shargo, Hector Moreno, Michael McKnight, Eileen McDonald, Wes Stewart, Evelyn Shiang, Ruth Ann Norton, Wendy C. Shields

**Affiliations:** 1 Johns Hopkins Center for Injury Research and Policy, Baltimore, MD USA; 2 Green and Healthy Homes Initiative, Baltimore, MD USA

**Keywords:** Injury prevention, Community intervention, Housing

## Abstract

**Background:**

Decades of research and practice experience have led to an extensive body of evidence about effective home safety modifications. However, the benefits of safety modifications have not reached all segments of society. Poor quality housing in low-income neighborhoods, along with limited access to safety products and injury prevention information, can be significant barriers to child safety.

**Methods:**

This is a longitudinal study of 300 low-income families in Baltimore City and Baltimore County with children under 7 years of age who are referred from existing Green & Healthy Homes Initiative (GHHI) home visiting programs. Three home visits will be completed to assess home injury hazards using a previously developed tool, the Children’s Housing Assessment for a Safe Environment (CHASE), and provide a Scope of Work that includes home modifications specific to the identified home injury hazards. An Assessor will also provide do-it-yourself education materials and injury prevention supplies to assist residents in completing the modifications. If the parent or caregiver is unable to complete the home modifications, a professional Housing Intervention Services team will complete the home modifications necessary to prevent injury in the home. This study will involve both quantitative and qualitative data analysis methods. Paired and regression analyses will be conducted to examine the maintenance of modifications and the variables associated with positive outcomes. A thematic analysis of staff and participant interviews will be used to identify perceived barriers and facilitators of successful program implementation.

**Discussion:**

Better data on residential injuries of children and an improvement in the overall surveillance of home injuries are necessitated. This study will set a strong foundation for a larger future study of health and cost effectiveness outcomes and will advance our understanding of the feasibility, costs, and potential benefits of addressing and preventing home injuries to children.

## Background

Unintentional injuries are the leading cause of death in children in the US, resulting in 12,000 deaths and 9.2 million emergency department visits each year (Centers for Disease Control and Prevention 2021). Children are especially at risk of injury in the home, with home-related injuries such as poisoning, suffocation, and drowning contributing substantially to injury-related death in this age group; an estimated 3,100 deaths occur annually due to home injury in children aged 14 and under alone (Centers for Disease Control and Prevention 2021; Centers for Disease Control and Prevention 2023). For every death, there are almost 1600 nonfatal home injuries, too many of which lead to lifelong disability or reduced quality of life for children and their families (National Safety Council 2023). Adding to these tragedies is that many of these injuries are preventable with proven and relatively simple home modifications (Turner et al. 2011).

Low-income and certain minority populations are disproportionately affected by child injuries, though racial disparities seen in injury rates can be attributed to living in dangerous environments with greater risk for injury and a host of social disparities rather than race or ethnicity (National Safety Council. 2023; Shenassa et al. 2017; Fallat et al. 2006; Bernard et al. 2007; Kendrick et al. 2009). Housing conditions in low-income neighborhoods likely contribute to low-income families’ increased risk for home injury for children and adults alike (Bishai et al. 2002; Reading et al. 2017; Lyons et al. 2006; Haynes et al. 2003; Reimers and Laflamme 2005). Residents of all ages living in substandard housing are at increased risk for fire, electrical injuries, lead poisoning, falls, and other injuries. Several studies have found that children living in socioeconomically disadvantaged neighborhoods are at increased risk of injury, even after accounting for individual-level factors (Reading et al. 2017; Haynes et al. 2003; Durkin et al. 1994; O’Campo et al. 2000; Parker et al. 2017).

Previous research on modifications to low-income housing has demonstrated that home modification interventions can reduce injury (Turner et al. 2011). A widely cited example of successfully modifying housing conditions to reduce child injury is New York City’s “Children Can’t Fly” program (Spiegel and Lindaman 1995), which installed window guards on high-rise apartments and is credited with significant reduction in morbidity and mortality due to falls from windows. The success of the program resulted in legislation requiring landlords to install window guards. Studies of smoke alarm canvassing and installation programs provide another successful example of modifying the home environment to reduce home injury risk to children (Gielen et al. 2013; Ta et al. 2006; Ballesteros et al. 2005; Wintemute et al. 2014). A comprehensive, internationally representative Cochrane review of interventions to reduce in-home hazards in households with children found that seven of the eight studies observed either a reduction in hazards or an increase in the presence of safety features (fireguards, electric socket covers, cabinet locks, window safety, and hot water temperature) (Parker et al. 2017). Interventions involving home safety modifications align well with the Health Belief Model (HBM), which suggests that individuals’ attitudes and beliefs about health problems influence their health-related behaviors. For example, installation programs reduce families’ perceived barriers to maintaining safe housing by overcoming limitations to accessing the needed safety products and services.

In 1976, the US government recognized the need for formal housing hazard assessment tools when the Centers for Disease Control and Prevention (CDC) and the American Public Health Association issued the Basic Housing Inspection Manual (Centers for Disease Control and Prevention 2009). Since that time, additional housing assessment tools and programs have emerged including United States Department of Housing and Urban Development’s (HUD) Housing Quality Standards (HQS) and, in 2005, the launch of the Healthy Homes Initiative, which includes the Healthy Housing Inspection Manual. Despite this progress, the lack of a single validated and comprehensive housing hazard assessment tool has been identified as a major gap in the fight against housing-related health hazards. Although there are separate standardized tools for specific indoor environmental hazards, such as lead paint, asbestos, and radon, there is no standardized tool covering the many different injury risks in the home, such as fire hazards, fall hazards, and medicine storage (Jacobs 2006). A literature review of housing and children’s health calls for better data on residential injuries of children, particularly emphasizing the need for an improvement in the overall surveillance of home injuries (Hood 2005). A validated and comprehensive injury risk assessment tool, therefore, would be an important contribution and could be utilized in the future as a key part of a comprehensive home hazard assessment, and for better assessments of home injury risk for children specifically. The Children’s Housing Assessment for a Safe Environment (CHASE) tool is one such assessment that systematically identifies injury hazards. As described in a previous article, the CHASE tool demonstrated significantly greater likelihood of detecting hazards in the homes of injured children compared to HUD’s HQS tool while uniquely assessing risks that are hazardous to children, such as furniture tipping and medicine storage (Shields et al. 2020).

The study objectives are to: (1) implement injury prevention measures based on safety hazards identified using the CHASE tool within 300 low-income households (at or below 80% Area Median Income) in Baltimore City or County; (2) determine the costs of the injury prevention measures completed by residents and/or professional providers; and (3) conduct a formative evaluation to determine the feasibility of incorporating injury prevention into residential programs from the perspective of installers and residents, including identifying barriers and facilitators, and understand the consistency of implementing and maintaining the injury prevention measures by residents and staff.

## Methods

### Study design and setting

The CHASE Implementation Study will use quantitative and qualitative methods and a non-experimental design to estimate the magnitude of home injury risks, identify strategies for addressing those risks and sustaining those interventions, and calculate the costs of housing-related modifications to prevent childhood home injuries. The Johns Hopkins Institutional Review Board has approved this study protocol. The study will enroll clients from families living in Baltimore City or Baltimore County receiving housing services from Green & Healthy Homes Initiative (GHHI)’s home visiting programs. GHHI is a Baltimore-based non-profit organization that coordinates funding from government and private institutions to deliver cost-effective services that assess and remediate a range of home health hazards. GHHI and JHU have partnered over the past several years to increase the inclusion of injury risk identification and modification in existing GHHI programs.

Participating families will first receive two home visits from a housing assessor using the CHASE tool to identify injury hazards in the home, outline a workplan for remediation, and assess the short-term maintenance of home modifications. On a third visit, study data collectors will conduct the home assessment. During each home visit, the participant will complete a questionnaire and receive feedback with results from the home inspection.

### Theoretical grounding

The foundation of the CHASE Implementation Study is grounded in the Health Belief Model (HBM), which suggests that individuals are most likely to change their behavior if they believe: (1) that they are at risk of a serious condition and (2) that making the recommended change will prevent them from acquiring that condition. The HBM was selected due to its focus on perceived risk and self-efficacy and because most of the items on the CHASE assessment can be addressed at the individual level, making the HBM an appropriate model. Of the six constructs of the HBM—perceived susceptibility, severity, benefits, and barriers; self-efficacy; and cues to action—the study team focused on five: perceived susceptibility, severity (combined to perceived risk), self-efficacy, and cues to action. We did not include content about barriers and benefits because those are addressed by the home modifications included project participation and in the interest of having the family feedback form being as concise as possible.

### Measures

#### Baseline measures

The baseline interview will be conducted over the phone and will document household and participant sociodemographic characteristics, home characteristics, and measures of housing stability.

#### CHASE Home Assessment Form and Family Feedback Form

The CHASE tool is a 35-item home injury hazard assessment organized into 15 subdomains (e.g., carbon monoxide risks, falling furniture, and hot water burns). Each item on the CHASE tool assesses whether a household passes or fails to address a household hazard, offers facts about the hazard, and gives user-friendly, nationally accepted best practices and information on how to remediate the hazards to make homes safer. For example, the medicine storage subdomain includes the item, “All prescription and over-the-counter medicines have childproof caps,” with the response options, “Yes” or “No.” For this project, the CHASE tool was adapted into the CHASE Home Assessment Form, which includes all elements of the CHASE tool but further allows the housing assessor to specify home modifications needed to remediate the hazards during home inspections.

The Family Feedback Form is a companion form that uses information transferred from the CHASE Assessment Form to provide participants a short description of each home injury risk being assessed and why it is important, whether the home passed or failed each item and why, and next steps to be taken. In some instances, the form recommends calling a contractor for changes that the parent or caregiver cannot complete on their own. The Family Feedback Form will guide the educational intervention that will be delivered with the goal of preparing the parent or caregiver to initiate recommended changes. For example, on the topic of poison storage, the housing assessor will give the participant a lock box or cabinet locks and show them how to install it. Accordant with the HBM, these instructions are intended to increase the self-efficacy of the participant by showing them how to install and/or use a home safety product. The “Why This is Important” column of the Family Feedback Form also contains a perceived risk and/or an efficacy message that will be reiterated to the participant to reinforce the messages by a credible individual.

#### Health belief questionnaire

The Health Belief Questionnaire will measure perceived risk and self-efficacy which will be self -administered during all three home visits. The questionnaire consists of 17 questions that the participant will respond to on a 5-point Likert scale, ranging from “strongly agree” to “strongly disagree.” Sample questions include “I believe my child could get injured in my home” and “I have no idea how to make changes in my home to make it safer, such as installing cabinet locks or securing furniture.” Responses collected from the Health Belief Questionnaire will inform an understanding of the participants’ attitudes toward home hazards and making recommended changes, which will be examined for potential effects on the adoption and maintenance of safety modifications in the home.

#### Follow-up survey

Each time the home assessment is repeated, the Follow-up Survey will be administered to measure the time and cost to complete home modifications as well as collect via interview qualitative descriptions of the participants’ experiences with the assessment and modifications to better understand barriers and facilitators of the implementation of the recommended modifications.

### Procedures

**Fig. 1:**
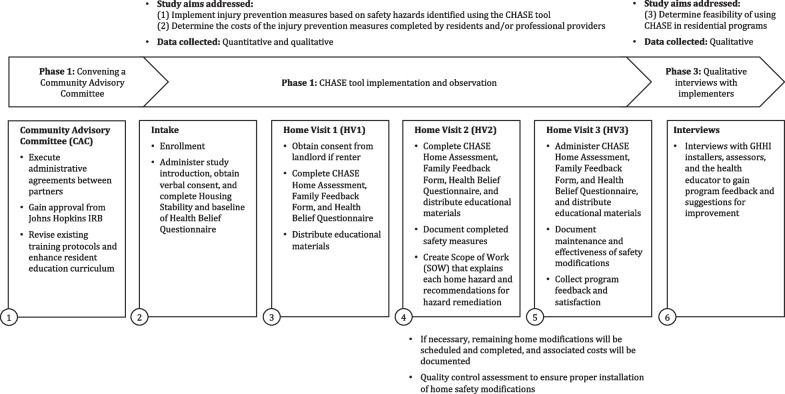
Diagram of study flow

The project is being conducted in three phases, the first of which has already been completed: (1) Convening a Community Advisory Committee (CAC); (2) CHASE tool implementation and observation; and (3) Qualitative interviews with implementers (See Fig. 1).

Phase 1 consisted of an initial convening of a CAC. CAC members were recruited from an existing Parent Advisory Board at the GHHI that offers feedback from family members. Parent Advisory Board members volunteered to participate and previously received program services from GHHI. We invited 11 Parent Advisory Board members to participate in the CAC. The CAC convened on August 23, 2021, with 6 members in attendance. CAC members were informed about the Implementation Study, reviewed key documents, and offered recommendations to ensure that those documents were clear and culturally appropriate for potential study participants. In advance of the meeting, CAC members were sent the oral consent form, Family Feedback Form, Mutual Service Agreement, and Follow-Up Questionnaire. Their ideas for revising and improving the documents were solicited at the meeting through a facilitated discussion. This process allowed the researchers to hear directly from CAC members and informed the subsequent changes that were made to those documents prior to use.

In Phase 1, educational fliers were also developed, including an overview flier that will be distributed to all participants during each home visit and topic-specific fliers for each of the injury hazards that homes could fail upon inspection using the CHASE Assessment Form. These fliers employ the HBM constructs to guide participants toward making suggested changes in their home. For example, if a participant’s water temperature registers above 120 degrees Fahrenheit, a flier on burn safety will be given stating that children have thinner skin than adults, which makes them more susceptible to burns from high water temperatures. Additionally, the researchers partnered with the creators of Make Safe Happen, a children’s home safety app, to help participants identify safety risks in their home and attain relevant products, such as a stair gate (McKenzie et al. 2018). A flier with the Make Safe Happen app information was developed to be provided to participants at the conclusion of the first home visit.

During Phase 2, 300 families will be enrolled. Parents or guardians (or emancipated minors who meet all other inclusion criteria)^1^ whose household is participating in a GHHI home visiting program will be invited to participate. Inclusion criteria include: (1) households with at least one child under 7 years of age or foster care families planning to host a child under 7; (2) the child under 7 years of age lives with the parent or legal guardian most of the time; (3) household income at or below 80% Area Median Income as defined by HUD; and (4) a home address in Baltimore City or Baltimore County.

While scheduling regular GHHI program visits, a GHHI Client Intake Specialist will use a project introduction script and eligibility screening form to briefly introduce the CHASE Implementation Study and review details, including a description of the project services and timeline, and determine if the parent or guardian is potentially interested and eligible to participate. Among those interested and eligible, the Client Intake Specialist will conduct the oral consent process, collect the baseline measures about the family, and schedule the first of three home visits, Home Visit 1 (HV1). Client intake specialist are bachelors trained as professionals whose roles requires customer service and project coordination skills.

HV1 will be scheduled within two weeks after intake. During HV1, a GHHI Assessor will visit the participant’s home, provide a copy of the completed consent form, and ask the study participant to complete a Health Belief Questionnaire. The Assessor will conduct a home inspection using the CHASE Home Assessment Form. After inspecting the residence, the Assessor will transfer assessment details onto the Family Feedback Form. Using this as a guide, the Assessor will then conduct a walk-through of the home providing advice and tailored education to assist caregivers in completing the identified home modifications or calling a contractor to do so on their own. The Assessor will also provide injury prevention supplies, a copy of the Family Feedback Form that lists the hazards identified in the home, and educational materials to assist the participants in completing the recommended modifications within 30 days. With the client’s consent, some photographs may be taken to document hazards identified during the housing assessment; no identifiable people or items will be captured in the phone. Participants may refuse without any negative repercussions.

Home Visit 2 (HV2) will occur approximately 30 days after HV1. A GHHI Assessor will return to reinspect the home, completing another CHASE Home Assessment Form to identify which hazardous item(s) the participants were able to modify on their own. They will complete a new Family Feedback Form to inform the family about the remaining risks in the home or to communicate that the home has passed all of the CHASE assessment items. A Research Assistant will administer the Health Belief Questionnaire and conduct a Follow-Up Survey.

For participants who were unable to address all hazards identified during HV1 (e.g., because the family could not or did not have sufficient means to address the hazards identified with home modifications alone or by calling a contractor), the Assessor will draft a Scope of Work for completion of the needed modifications and arrange a time to return to the home with the GHHI Housing Intervention Services Team, a professional home modification team who will complete the installation of any incomplete home modifications as described in the Scope of Work.^2^ When modifications are needed outside the capacity of the Housing Intervention Services team, GHHI will arrange for a contractor to complete the Scope of Work. All costs for modifications whether by the GHHI home modification team or by outside contractors will be paid for by the project with the grant funding. As is standard procedure for GHHI, there will be a post-intervention quality control assessment conducted by an Assessor immediately after the injury prevention intervention to verify that all measures on the Scope of Work were installed properly and meet HUD and project standards. Any discrepancies will be resolved. GHHI will document all costs, both labor and supplies, associated with completing the Scope of Work.

Home Visit 3 (HV3) will occur three months after all safety modifications have been completed. During HV3, a Research Assistant will complete the CHASE Home Assessment Form, the Health Belief Questionnaire, and a second Follow-Up Survey to assess the maintenance and effectiveness of the modifications. The Family Feedback Form will also be completed to inform families of any newly identified or persisting injury hazards, and informational resources will be provided to assist families in addressing identified risks. With the participant’s consent, additional photographs may be taken to demonstrate hazards identified as well as examples of successful repairs. These photographs will be used only for training purposes. We will make sure there are no identifiable people or items in the photographs. The participant can refuse to have photographs taken in their home.

The final phase of the study will involve qualitative interviews with those GHHI staff (Client Intake Specialist, Assessor, and Housing Intervention Services team) who are knowledgeable and experienced with the CHASE program to assess installer perspectives of feasibility, barriers, and facilitators to incorporating injury prevention measures into home visiting programs and sustaining those measures over time. GHHI will provide a list of staff involved in the project to the research team, and the study investigator will email each key informant to invite them to participate in a 30 min one-one-one interview.

### Sample size

A total of 300 homes (total of 900 home visits) will be studied in Baltimore City and Baltimore County, which will be referred from GHHI’s existing housing programs: Using a paired analysis of the number of hazards at Time 1 (HV1) versus Time 2 (HV3) with *N* = 300, *α* = 0.05, and assuming standard deviation 8.4 (Posner et al. 2004), we will have 80% power to detect a change in the mean number of hazards of 1.36.

### Data analysis plan

Descriptive statistics and frequency tabulations will be generated on the number of hazards per home at each time point and the total time and cost per home for modifications. Pass scores for the home (number of items passed/total items) will be calculated as well as the prevalence of each individual hazard (out of *N* = 300). The mean, median, and range of costs per household and per injury prevention measure will be calculated. Homes with outliers for the number, duration, and cost of modifications will be identified and the characteristics associated with those homes will be noted. A paired analysis will be used to test the change in mean number of hazards present between HV1 and HV3. Additional analyses will be performed for individual hazards and modifications.

Maintenance of modifications is of particular interest, along with variables that are associated with higher rates of maintenance, which will be examined by comparing HV2 and HV3 data; this will be an analysis of proportion of modifications still in place at HV3 by outcome variable and by modification options. Frequency distributions for the additional variables of interest (sociodemographic characteristics, characteristics of the homes, participant knowledge, participant capacity to complete home modifications, participant satisfaction with the program, and perceived barriers and facilitators collected during the initial home visit by the Assessor) will be prepared. These variables will be used to conduct regression analyses to explain the hazard and maintenance outcomes described above as a function of referral source, sociodemographic characteristics, housing, knowledge, installer (homeowner/tenant and GHHI), and satisfaction. To explore the capacity of caregivers to install injury prevention measures, paired data from HV1 and HV2 will be used to compute change in mean number of hazards.

For the qualitative analysis, open-ended questions on the participant Follow-Up Surveys and key informant interviews of GHHI staff will be used to develop a codebooks based on the content of the participant follow-up surveys and key informant interviews. Thematic analysis will be utilized to code data, a process common in qualitative research. Separate thematic analysis will be completed for each group. Through that process, perceived barriers and facilitators to implementation and sustainability will be explained, and participants’ suggestions for improvements will be identified and highlighted in the results. Resulting themes that are responsive to the objectives will be reported using illustrative quotes. The analysis will highlight implications for improved implementation of the CHASE tool and hazard remediation procedures from the perspectives of residents and the installers. Additionally, we expect at least some emergent themes to highlight potential processes underlying the trends captured in our quantitative data analysis. These results will be reported to contextualize our quantitative findings and reinforce or qualify results suggesting relationships between certain variables of interest and the successful implementation of home modifications.

## Discussion

Home injuries lead to 3100 deaths among children under the age of 15 every year (National Safety Council 2023). Despite this, we lack data on specific assessments of home injury risks for children. This study will generate information on known causes of unintentional home injuries to children that can then be used to advance our understanding of the feasibility, costs, and potential benefits of implementing effective preventative modifications.

The project will also produce user-friendly information on how to remediate the hazards in ways that can be easily integrated into multiple, ongoing home visiting programs and replicated nationally. From the study, we will learn how to promote equity by empowering parents and caregivers through education on maintaining healthy and safe housing that is free from known injury hazards for children. It is also anticipated that this project may generate the initial economic evidence after performing a program cost analysis which is needed to begin building the business case for sustainable Medicaid funding and private healthcare investments to fund household injury prevention services. This implementation study will set a strong foundation for a larger future study designed to measure the extent to which education, assessment, and home modifications improve health outcomes and reduce medical costs related to home injury.

The qualitative information collected in the study will help us understand the barriers and facilitators to implementing and maintaining home safety modifications in low-income populations. Given that low-income families are more likely to live in older homes with greater risks (Swope and Hernández 2019), the qualitative data from families with limited finances will help us understand what assistance is needed in order to moderate the structural issues and increased risks inherent in older homes.

This study is subject to limitations. First, our procedures which provide 30 days for the families to complete repairs on their own followed by a reinspection and then modifications provided by GHHI limit our ability to know what modifications families would make on their own without knowing that GHHI would return to make the modifications. We designed it this way with the hopes of learning what families could modify easily on their own to inform expectations and cost estimates for future work. We recognize that our estimates will be conservative. It is additionally important to acknowledge that this study does not utilize an experimental design. Although the standardization of the CHASE assessment promotes strong internal validity, the limited data produced from a pediatric population in one geographic area limit the generalizability of the data. Future work should utilize the tool in other programs that have access to the homes of young children, such as pediatric home visiting programs including those funded by Title V Maternal and Child Health Block Grants, which serve 140,000 households per year (Health Resources Services Administration 2022). In addition, future work should consider targeting housing risks beyond the pediatric population. Previous work from the Community Aging in Place—Advancing Better Living for Elders (CAPABLE) program, demonstrated a reduction in older adult falls through the delivery of a comprehensive program which included a handyman to address fall risks in the home (Szanton et al. 2014). Translating the comprehensive injury measures included on the CHASE tool to older adults could assist with aiding older adults to age in place. Further, it is unclear how overall housing quality will mediate the benefit of the CHASE measures. Therefore, after the completion of this implementation study there will still be the need to conduct either an experimental (e.g., randomized trial) or quasi-experimental (e.g., matched case–control) outcome study to determine the efficacy and cost effectiveness in reducing unintentional injuries in the home. Upon completing this implementation study, we will better understand the necessary steps for large scale adoption and implementation of the CHASE assessment and modification protocols in existing housing inspection programs and home visiting programs.

## Data Availability

Not applicable.

## Abbreviations

CDC: Centers for Disease Control and Prevention
HUD: United States Department of Housing and Urban Development
GHHI: Green & Healthy Homes Initiative
CHASE: Children’s Housing Assessment for a Safe Environment
HQS: Housing Quality Standards
CAC: Community Advisory Committee
HV1: Home Visit 1
HV2: Home Visit 2
HV3: Home Visit 3
MSA: Mutual Service Agreement
HBM: Health Belief Model
CAPABLE: Community Aging in Place—Advancing Better Living for Elders

## Footnotes

1 Henceforth, the term “parent or guardian” is inclusive of emancipated minors who meet all inclusion criteria.

2 Before any home modification is performed on a renter occupied dwelling, GHHI staff will contact the landlord via phone or email and have a Mutual Service Agreement (MSA) executed prior to allowing the home modifications to be installed in the home by the GHHI Housing Intervention Services team. Staff will provide program information, goals, and the type of work that could be performed in the home. Once verbal permission is provided, staff will email the landlord for agreement and a signature. The MSA includes language providing GHHI permission to perform work. Upon receipt of the signed MSA, the process will begin to complete home repairs.
